# Mortality Risk Among Patients With COVID-19 Prescribed Selective Serotonin Reuptake Inhibitor Antidepressants

**DOI:** 10.1001/jamanetworkopen.2021.33090

**Published:** 2021-11-15

**Authors:** Tomiko Oskotsky, Ivana Marić, Alice Tang, Boris Oskotsky, Ronald J. Wong, Nima Aghaeepour, Marina Sirota, David K. Stevenson

**Affiliations:** 1 Department of Pediatrics, University of California, San Francisco; 2 Bakar Computational Health Sciences Institute, University of California, San Francisco; 3 Department of Pediatrics, Stanford University School of Medicine, Stanford, California; 4 Graduate Program in Bioengineering, University of California, San Francisco; 5 Department of Anesthesiology, Perioperative and Pain Medicine, Stanford University School of Medicine, Stanford, California; 6 Department of Biomedical Data Science, Stanford University School of Medicine, Stanford, California; 7 Center for Academic Medicine, Stanford University School of Medicine, Stanford, California

## Abstract

**Importance:**

Antidepressant use may be associated with reduced levels of several proinflammatory cytokines suggested to be involved with the development of severe COVID-19. An association between the use of selective serotonin reuptake inhibitors (SSRIs)—specifically fluoxetine hydrochloride and fluvoxamine maleate—with decreased mortality among patients with COVID-19 has been reported in recent studies; however, these studies had limited power due to their small size.

**Objective:**

To investigate the association of SSRIs with outcomes in patients with COVID-19 by analyzing electronic health records (EHRs).

**Design, Setting, and Participants:**

This retrospective cohort study used propensity score matching by demographic characteristics, comorbidities, and medication indication to compare SSRI-treated patients with matched control patients not treated with SSRIs within a large EHR database representing a diverse population of 83 584 patients diagnosed with COVID-19 from January to September 2020 and with a duration of follow-up of as long as 8 months in 87 health care centers across the US.

**Exposures:**

Selective serotonin reuptake inhibitors and specifically (1) fluoxetine, (2) fluoxetine or fluvoxamine, and (3) other SSRIs (ie, not fluoxetine or fluvoxamine).

**Main Outcomes and Measures:**

Death.

**Results:**

A total of 3401 adult patients with COVID-19 prescribed SSRIs (2033 women [59.8%]; mean [SD] age, 63.8 [18.1] years) were identified, with 470 receiving fluoxetine only (280 women [59.6%]; mean [SD] age, 58.5 [18.1] years), 481 receiving fluoxetine or fluvoxamine (285 women [59.3%]; mean [SD] age, 58.7 [18.0] years), and 2898 receiving other SSRIs (1733 women [59.8%]; mean [SD] age, 64.7 [18.0] years) within a defined time frame. When compared with matched untreated control patients, relative risk (RR) of mortality was reduced among patients prescribed any SSRI (497 of 3401 [14.6%] vs 1130 of 6802 [16.6%]; RR, 0.92 [95% CI, 0.85-0.99]; adjusted *P* = .03); fluoxetine (46 of 470 [9.8%] vs 937 of 7050 [13.3%]; RR, 0.72 [95% CI, 0.54-0.97]; adjusted *P* = .03); and fluoxetine or fluvoxamine (48 of 481 [10.0%] vs 956 of 7215 [13.3%]; RR, 0.74 [95% CI, 0.55-0.99]; adjusted *P* = .04). The association between receiving any SSRI that is not fluoxetine or fluvoxamine and risk of death was not statistically significant (447 of 2898 [15.4%] vs 1474 of 8694 [17.0%]; RR, 0.92 [95% CI, 0.84-1.00]; adjusted *P* = .06).

**Conclusions and Relevance:**

These results support evidence that SSRIs may be associated with reduced severity of COVID-19 reflected in the reduced RR of mortality. Further research and randomized clinical trials are needed to elucidate the effect of SSRIs generally, or more specifically of fluoxetine and fluvoxamine, on the severity of COVID-19 outcomes.

## Key Points

**Question:**

Are selective serotonin reuptake inhibitors (SSRIs), specifically fluoxetine hydrochloride, associated with a lower mortality risk among patients with COVID-19?

**Findings:**

In this multicenter cohort study analyzing electronic health records of 83 584 patients diagnosed with COVID-19, including 3401 patients who were prescribed SSRIs, a reduced relative risk of mortality was found to be associated with the use of SSRIs—specifically fluoxetine—compared with patients who were not prescribed SSRIs.

**Meaning:**

These findings suggest that SSRI use may reduce mortality among patients with COVID-19, although they may be subject to unaccounted confounding variables; further investigation via large, randomized clinical trials is needed.

## Teaser

This cohort study investigates whether use of selective serotonin reuptake inhibitors is associated with a reduction in the relative risk of mortality for patients with COVID-19.

## Introduction

As the world searches for effective treatments for COVID-19, evidence from recent studies has suggested that selective serotonin reuptake inhibitor (SSRI) antidepressants may be of benefit.^1,2,3,4,5,6^ The severe respiratory illness of COVID-19 is primarily triggered by an intense proinflammatory host response.^7^ Selective serotonin reuptake inhibitors are one of the most prescribed, widely available classes of antidepressants used for treating psychological conditions, including depression and anxiety disorders.^8,9,10^ It has been previously observed that SSRIs may have anti-inflammatory properties mediated through a reduction of several proinflammatory cytokines, including interleukin 6 and tumor necrosis factor.^11,12^ Selective serotonin reuptake inhibitors may also be beneficial to patients with COVID-19 through their inhibiting effect on the acid sphingomyelinase/ceramide system, which may have an important role in SARS-CoV-2 infection.^13,14,15^ In fact, an intake of functional inhibitors of acid sphingomyelinase activity medications, which inhibit the acid sphingomyelinase/ceramide system and include fluoxetine hydrochloride and fluvoxamine maleate (among other medications), was associated with substantially reduced likelihood of intubation or death among hospitalized patients with COVID-19.^16,17^

Although findings from some studies have suggested that psychiatric diagnosis, including mood and anxiety disorders, may be an independent risk factor for COVID-19 infection,^18,19^ other epidemiological studies have suggested that major depression could be associated with reduced mortality in COVID-19,^20,21^ possibly owing to the use of antidepressants in this population.^1,20^ A recent observational study^1^ showed a decreased mortality rate in hospitalized patients with COVID-19 who were prescribed antidepressants (n = 460), in particular SSRIs, but this study had limited power. Moreover, a small, randomized clinical trial^2^ (n = 152) indicated a decrease in disease severity in patients with COVID-19 given the SSRI fluvoxamine. However, the follow-up duration for this study was short (30 days); therefore, the outcomes of patients beyond this time frame are not known. Another small, prospective clinical trial^3^ (n = 113) found that patients who received early treatment with fluvoxamine were not hospitalized or did not have residual symptoms, unlike untreated patients; however, this study also had a short duration of follow-up (14 days) and, as an open-label trial, was subject to potential biases. Preclinical studies have demonstrated the antiviral activity of fluoxetine, an SSRI with properties similar to those of fluvoxamine, against SARS-CoV-2–infected Vero E6 cells^4^ and human lung tissue.^5^ A study comparing differential gene expression signatures from drug-treated cell lines with those from genetic knockdown of select cytokine storm–related inflammatory genes^6^ found greater concordance in these signatures with fluoxetine than with dexamethasone, a corticosteroid used in treating patients with COVID-19. Thus, we investigated the hypothesis that SSRIs, and specifically fluoxetine and fluvoxamine, are associated with a reduction in the relative risk (RR) of mortality for patients with COVID-19 by using a large electronic health record (EHR) database consisting of a diverse population of nearly half a million patients with COVID-19 across the US.

## Methods

The study was approved by the University of California, San Francisco, institutional review board, which considered this work secondary research for which consent is not required. This study followed the Strengthening the Reporting of Observational Studies in Epidemiology (STROBE) reporting guideline.^22^

Data from the Cerner Real World Data COVID-19 deidentified EHR database containing records of 490 373 patients with COVID-19 across 87 health care centers were analyzed. The Cerner Real World Data COVID-19 database represents patients with a diagnosis of COVID-19 or COVID-19 exposure who had an emergency department or urgent care visit or were admitted for observation or hospitalized. Encounters include laboratory and pharmacy information (eg, medication orders, dispensing), which are date and time stamped. Only patients with COVID-19 diagnosis confirmed by a laboratory test for SARS-CoV-2 (nucleic acid amplification tests and immunoassays) and/or by *International Statistical Classification of Diseases and Related Health Problems, Tenth Revision* (*ICD-10*) code U07.1 (for COVID-19 confirmed by laboratory testing),^23^ with known demographic characteristics (age, sex, and race and ethnicity), and who were 18 years or older from January to September 2020 were included in our study (n = 90 834) (Figure). Patients with COVID-19 and a medication order for an SSRI with an order status of active or completed and without a designation of as needed (ie, medication taken only when needed) at least once within a period of 10 days before and 7 days after their first recorded COVID-19 diagnosis were compared with patients with COVID-19 and no SSRI orders. We excluded patients with other order status (eg, inactive, discontinued, or unknown). Individuals prescribed SSRIs outside this period were excluded (n = 7250), resulting in a final inclusion of 83 584 patients (Figure). The following SSRIs were included: escitalopram oxalate, paroxetine hydrochloride, paroxetine mesylate, sertraline hydrochloride, fluoxetine, citalopram hydrobromide, vortioxetine hydrobromide, fluvoxamine, and vilazodone hydrochloride. Subgroups exclude patients for whom more than 1 of the different SSRIs was ordered during the inclusion period. Considered comorbidities, identified by using *International Classification of Diseases, Ninth Revision, Clinical Modification* (*ICD-9-CM*), and *International Statistical Classification of Diseases, Tenth Revision, Clinical Modification* (*ICD-10-CM*), diagnosis codes, included hypertension (I10-I16 and 401.X-405.X), diabetes (O24, E11, E10, E13, and 250.X), chronic obstructive pulmonary disease (J44, 491.2, 493.2, and 496.X), obesity (E66.0, E66.1, E66.2, E66.8, E66.9, 278.00, 278.01, 278.03, Z68.3, Z68.4, V85.3, and V85.4), heart conditions (I20.X, I21.X, I22.X, I23.X, I24.X, I25.X, I42.X, I50.X, 410.X, 411.X, 412.X, 413.X, 414.X, 428.X, and 425.X), cerebrovascular disease (I6X.X, G45.X, G46.X, and 43X.X), cancer (C00-C96, 14X.X, 15X.X, 16X.X, 17X.X, 18X.X, 19X.X, and 20X.X), and chronic kidney disease (N18.X and 585.X). Prescription indications, identified by using *ICD-9-CM* and *ICD-10-CM* codes, included mood and anxiety disorders (F30.X-F39.X, F40.X-F48.X, 296.X, 300.X, 309.X, and 311.X) and other psychiatric disorders not mood or anxiety disorders (F0X.X, F1X.X, F2X.X, F5X.X-F9X.X, 290.X-295.X, 297.X-299.X, 301.X-308.X, 310.X, and 312.X-316.X). Body mass index (calculated as weight in kilograms divided by height in meters squared) values were identified by Logical Observation Identifiers Names and Codes 39156-5 and 89270-3. Body mass index values of at least 30 were used to confirm the diagnosis of obesity by *ICD-9-CM* or *ICD-10-CM* code. Primary outcome was death after the onset of COVID-19.

**Figure.**
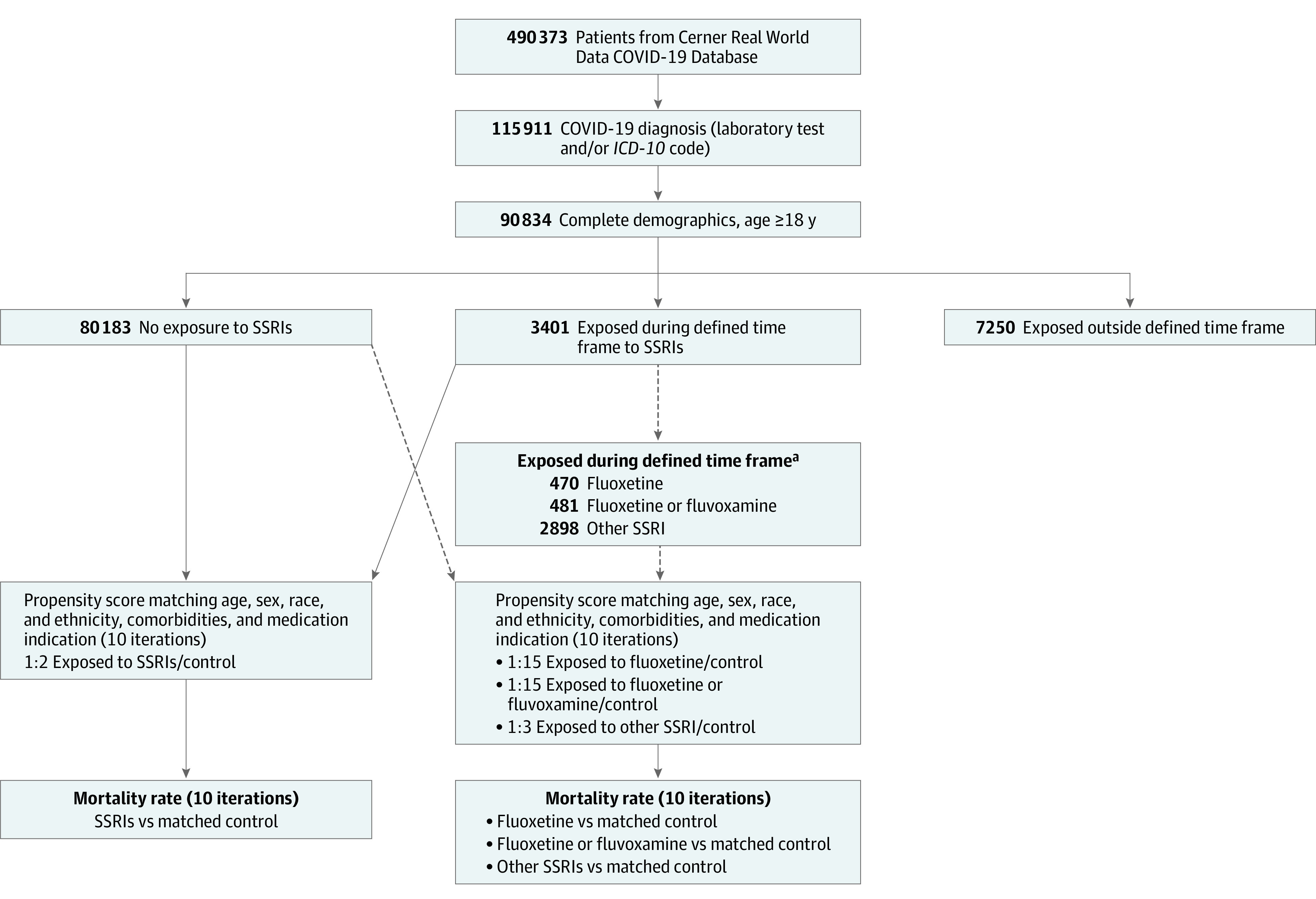
Flow Diagram of Patient Selection and Analysis *ICD-10* indicates *International Statistical Classification of Diseases and Related Health Problems, Tenth Revision*; SSRI, selective serotonin reuptake inhibitor. ^a^Among the 3401 who received an SSRI, 470 received fluoxetine only and 11 received fluvoxamine only (481 patients received either fluoxetine or fluvoxamine) and 2898 received an SSRI other than fluoxetine or fluvoxamine.

### Statistical Analysis

The R Matchit package, version 3.0.2 (R Program for Statistical Computing) was used to perform propensity score matching with a nearest-neighbor method to match SSRI-treated patients with control patients (1:2 ratio), fluoxetine-treated patients with control patients (1:15 ratio), fluoxetine- or fluvoxamine-treated patients with control patients (1:15 ratio), and patients treated with an SSRI other than fluoxetine or fluvoxamine (citalopram, escitalopram, paroxetine, sertraline, vortioxetine, or vilazodone) with control patients (1:3 ratio). The propensity score was estimated using logistic regression of the treatment based on demographic characteristics (age, sex, and race and ethnicity), the encounter type at the time of the first recorded COVID-19 diagnosis, comorbidities (hypertension, diabetes, chronic obstructive pulmonary disease, obesity, heart conditions, cerebrovascular disease, cancer, and chronic kidney disease), and SSRI prescription indications (mood or anxiety disorder, other psychiatric disorder [not mood or anxiety disorder]) with age at encounter as a continuous variable, and the remaining as categorical variables. To assess covariate balance, density plots of the distribution of propensity scores for the treated and control groups before and after matching were created, and standardized mean differences between cohorts before and after propensity score matching were calculated.

We performed the Welch 2-sample, 2-sided *t* test for continuous variables and the Pearson χ^2^ test with Yates continuity correction for categorical variables to evaluate whether there was a significant difference when comparing the 2 groups. We performed 10 iterations and evaluated mortality rate. Relative risks with 95% CIs were calculated for each iteration. Each iteration included all SSRI-treated patients and a subset of control patients chosen by propensity score matching. No SSRI-treated patients were discarded. We compared patients treated with (1) an SSRI, (2) specifically the SSRI fluoxetine, (3) specifically the SSRIs fluoxetine or fluvoxamine, and (4) SSRIs other than fluoxetine or fluvoxamine with their matched control patients. Owing to the large number of control patients, several of these patients could have had the same propensity score value, and therefore some SSRI-treated patients could be matched to several more than the number of control patients specified by the ratio (2, 3, or 15) having the same propensity score. To explore this variability and show the robustness of the results, we performed bootstrapping,^24^ with 10 iterations for each comparison, by varying the control patients who had a tie in their propensity scores. A significance threshold of .05 was applied to Benjamini-Hochberg–corrected *P* values. The mean fluoxetine-equivalent dose prescribed for SSRI-treated patients was calculated for each comparison.^25,26^

## Results

Among the 83 584 eligible patients, 3401 (2033 women [59.8%] and 1368 men [40.2%]; mean [SD] age, 63.8 [18.1] years) had an order for an SSRI in the defined time frame, with 470 (280 women [59.6%] and 190 men [40.4%]; mean [SD] age, 58.5 [18.1] years) receiving fluoxetine only, 11 (5 women [45.5%] and 6 men [54.5%]; mean [SD] age, 62.9 [11.2] years) receiving fluvoxamine only, 481 (285 women [59.3%] and 196 men [40.7%]; mean [SD] age, 58.7 [18.0] years) receiving fluoxetine or fluvoxamine, and 2898 (1733 women [59.8%] and 1165 men [40.2%]; mean [SD] age, 64.7 [18.0] years) receiving other SSRIs. The remaining 80 183 patients had no history of SSRI exposure (control patients). Cohort characteristics are shown in Table 1, Table 2, and eTables 1 to 4 in the Supplement. Propensity score matching was performed, and the propensity score distributions and standardized mean differences of covariates between treated and control groups before and after matching showed adequate balance after matching, with the absolute value of standardized mean difference of less than 0.1 for all covariates after matching (eFigures 1-4 and eTables 1-4 in the Supplement).

**Table 1.** Cohort Demographic Characteristics Before Propensity Score Matching With SMDs for Patients Prescribed Any SSRI or a Specific SSRI Compared With Control Patients Not Treated With an SSRI^a^

Characteristic	SSRI exposure																		
	None, No. (%) (n = 80 183)	Any (n = 3401)		Citalopram (n = 533)		Escitalopram (n = 930)		Fluoxetine (n = 470)		Fluvoxamine (n = 11)		Paroxetine (n = 263)		Sertraline (n = 1121)		Vilazodone (n = 24)		Vortioxetine (n = 27)	
		Data	SMD, %	Data	SMD, %	Data	SMD, %	Data	SMD, %	Data	SMD, %	Data	SMD, %	Data	SMD, %	Data	SMD, %	Data	SMD, %
Age, mean (SD), y	51.6 (19.2)	63.8 (18.1)	−0.66	67.1 (16.1)	−0.88	64.0 (18.5)	−0.66	58.5 (18.1)	−0.38	62.9 (11.2)	−0.72	64.6 (15.7)	−0.74	64.4 (18.7)	−0.68	56.2 (16.8)	−0.26	59.5 (19.9)	−0.41
Age, y																			
18-39	24 909 (31.1)	417 (12.3)	0.47	34 (6.4)	−0.67	126 (13.5)	0.43	83 (17.7)	0.32	0	0.95	18 (6.8)	−0.65	143 (12.8)	0.45	4 (16.7)	0.34	5 (18.5)	0.29
40-49	12 415 (15.5)	291 (8.6)	−0.21	37 (6.9)	−0.27	72 (7.7)	−0.24	46 (9.8)	−0.17	2 (18.2)	−0.07	24 (9.1)	−0.19	101 (9.0)	−0.20	6 (25.0)	−0.24	3 (11.1)	0.13
50-59	13 979 (17.4)	521 (15.3)	0.06	80 (15.0)	0.07	138 (14.8)	0.07	95 (20.2)	−0.07	1 (9.1)	−0.25	50 (19.0)	−0.04	145 (12.9)	0.13	4 (16.7)	0.02	5 (18.5)	−0.03
60-69	12 370 (15.4)	701 (20.6)	−0.14	126 (23.6)	−0.21	174 (18.7)	−0.09	102 (21.7)	−0.16	4 (36.4)	−0.49	64 (24.3)	−0.23	218 (19.4)	−0.11	4 (16.7)	−0.03	5 (18.5)	−0.08
70-79	9340 (11.6)	712 (20.9)	−0.25	122 (22.9)	−0.30	197 (21.2)	−0.26	83 (17.7)	−0.17	4 (36.4)	−0.60	59 (22.4)	−0.29	233 (20.8)	−0.25	3 (12.5)	−0.03	4 (14.8)	−0.09
≥80	7168 (8.9)	759 (22.3)	0.38	134 (25.1)	0.44	223 (24.0)	0.41	61 (13.0)	0.13	0	0.44	48 (18.3)	0.27	281 (25.1)	0.44	3 (12.5)	0.12	5 (18.5)	0.28
Sex																			
Female	39 889 (49.7)	2033 (59.8)	−0.20	333 (62.5)	−0.26	564 (60.6)	−0.22	280 (59.6)	−0.20	5 (45.5)	0.09	154 (58.6)	−0.18	652 (58.2)	−0.17	13 (54.2)	−0.09	17 (63.0)	−0.27
Male	40 292 (50.3)	1368 (40.2)	0.20	200 (37.5)	0.26	366 (39.4)	0.22	190 (40.4)	0.20	6 (54.5)	−0.09	109 (41.4)	0.18	469 (41.8)	0.17	11 (45.8)	0.09	10 (37.0)	0.27
Race																			
American Indian or Alaska Native	1684 (2.1)	43 (1.3)	0.07	6 (1.1)	0.08	11 (1.2)	0.07	4 (0.9)	0.10	0	0.21	3 (1.1)	0.08	19 (1.7)	0.03	0	0.21	0	0.21
Asian or Pacific Islander	1982 (2.5)	40 (1.2)	0.10	5 (0.9)	0.12	14 (1.5)	0.07	4 (0.9)	0.13	0	0.23	3 (1.1)	0.10	13 (1.2)	0.10	1 (4.2)	−0.10	0	0.23
Black or African American	16 339 (20.4)	478 (14.1)	0.17	89 (16.7)	0.10	105 (11.3)	0.25	78 (16.6)	0.10	1 (9.1)	−0.32	38 (14.4)	0.16	161 (14.4)	0.16	0	0.72	1 (3.7)	−0.53
White	48 981 (61.1)	2602 (76.5)	−0.34	399 (74.9)	−0.30	733 (78.8)	−0.39	349 (74.3)	−0.28	8 (72.7)	−0.25	194 (73.8)	−0.27	855 (76.3)	−0.33	22 (91.7)	−0.77	25 (92.6)	−0.81
Mixed	331 (0.4)	8 (0.2)	0.03	1 (0.2)	0.04	1 (0.1)	0.06	0	0.09	0	0.09	2 (0.8)	−0.05	4 (0.4)	0.01	0	0.09	0	0.09
Other	10 864 (13.5)	230 (6.8)	−0.23	33 (6.2)	−0.25	66 (7.1)	−0.21	35 (7.4)	−0.20	2 (18.2)	−0.13	23 (8.7)	−0.15	69 (6.2)	−0.25	1 (4.2)	−0.34	1 (3.7)	−0.36
Ethnicity																			
Hispanic or Latino	35 575 (44.4)	972 (28.6)	0.33	148 (27.8)	0.35	295 (31.7)	0.26	117 (24.9)	0.42	1 (9.1)	−0.87	95 (36.1)	0.17	297 (26.5)	0.38	4 (16.7)	0.63	11 (40.7)	0.07
Not Hispanic or Latino	44 606 (55.6)	2429 (71.4)	−0.33	385 (72.2)	−0.35	635 (68.3)	−0.26	353 (75.1)	−0.42	10 (90.9)	−0.87	168 (63.9)	−0.17	824 (73.5)	−0.38	20 (83.3)	−0.63	16 (59.3)	−0.07

Abbreviations: SMD, standardized mean difference; SSRI, selective serotonin reuptake inhibitor.

^a^ Unless otherwise indicated, data are expressed as number (%) of patients.

**Table 2.** Cohort Clinical Characteristics Before Propensity Score Matching With SMDs for Patients Prescribed Any SSRI or a Specific SSRI Compared With Control Patients Not Treated With an SSRI^a^

Characteristic	SSRI exposure																		
	None, No. (%) (n = 80 183)	Any (n = 3401)		Citalopram (n = 533)		Escitalopram (n = 930)		Fluoxetine (n = 470)		Fluvoxamine (n = 11)		Paroxetine (n = 263)		Sertraline (n = 1121)		Vilazodone (n = 24)		Vortioxetine (n = 27)	
		Data	SMD, %	Data	SMD, %	Data	SMD, %	Data	SMD, %	Data	SMD, %	Data	SMD, %	Data	SMD, %	Data	SMD, %	Data	SMD, %
Encounter type																			
Inpatient	35 320 (44.1)	2515 (73.9)	−0.64	404 (75.8)	−0.69	693 (74.5)	−0.65	318 (67.7)	−0.49	10 (90.9)	−1.16	203 (77.2)	−0.72	838 (74.8)	−0.66	18 (75.0)	−0.66	16 (59.3)	−0.31
Emergency	40 759 (50.8)	647 (19.0)	0.71	93 (17.4)	0.75	168 (18.1)	0.74	107 (22.8)	0.61	0	1.44	44 (16.7)	0.77	218 (19.4)	0.70	6 (25.0)	0.55	8 (29.6)	0.44
Observation	3028 (3.8)	220 (6.5)	−0.12	33 (6.2)	−0.11	60 (6.5)	−0.12	44 (9.4)	−0.23	1 (9.1)	−0.22	15 (5.7)	−0.09	60 (5.4)	−0.08	0	0.28	3 (11.1)	0.28
Urgent care	1074 (1.3)	19 (0.6)	0.08	3 (0.6)	0.08	9 (1.0)	0.04	1 (0.2)	0.13	0	0.17	1 (0.4)	0.10	5 (0.4)	0.10	0	0.17	0	0.17
Condition																			
Obesity	30 003 (37.4)	1327 (39.0)	−0.03	219 (41.1)	−0.08	367 (39.5)	−0.04	191 (40.6)	−0.07	5 (45.5)	−0.16	100 (38.0)	−0.01	416 (37.1)	0.01	12 (50.0)	−0.26	11 (40.7)	−0.07
Cancer	4200 (5.2)	255 (7.5)	−0.09	51 (9.6)	−0.17	67 (7.2)	−0.08	23 (4.9)	0.02	1 (9.1)	−0.15	11 (4.2)	0.05	98 (8.7)	−0.14	1 (4.2)	0.05	1 (3.7)	0.07
CVD	5797 (7.2)	534 (15.7)	0.27	97 (18.2)	0.33	146 (15.7)	0.27	62 (13.2)	0.20	4 (36.4)	0.75	34 (12.9)	0.19	183 (16.3)	0.29	3 (12.5)	0.18	2 (7.4)	−0.01
Chronic kidney disease	9891 (12.3)	727 (21.4)	−0.24	123 (23.1)	−0.28	194 (20.9)	−0.23	89 (18.9)	−0.18	2 (18.2)	−0.16	54 (20.5)	−0.22	250 (22.3)	−0.27	1 (4.2)	−0.30	7 (25.9)	−0.35
COPD	6059 (7.6)	599 (17.6)	0.31	95 (17.8)	0.31	155 (16.7)	0.28	92 (19.6)	0.36	2 (18.2)	0.32	41 (15.6)	0.25	196 (17.5)	0.30	4 (16.7)	0.28	6 (22.2)	0.42
Diabetes	24 439 (30.5)	1448 (42.6)	−0.25	228 (42.8)	−0.26	382 (41.1)	−0.22	182 (38.7)	−0.17	4 (36.4)	−0.13	115 (43.7)	−0.28	507 (45.2)	−0.31	7 (29.2)	0.03	14 (51.9)	−0.45
Heart disease	15 526 (19.4)	1232 (36.2)	−0.38	212 (39.8)	−0.46	326 (35.1)	−0.36	151 (32.1)	−0.30	4 (36.4)	−0.39	90 (34.2)	−0.34	423 (37.7)	−0.42	7 (29.2)	−0.23	9 (33.3)	−0.32
Hypertension	37 243 (46.4)	2406 (70.7)	−0.51	390 (73.2)	−0.57	643 (69.1)	−0.47	322 (68.5)	−0.46	8 (72.7)	−0.56	196 (74.5)	−0.60	800 (71.4)	−0.52	15 (62.5)	−0.33	17 (63.0)	−0.34
Mood or anxiety disorder	14 703 (18.3)	2150 (63.2)	−1.03	329 (61.7)	−0.99	575 (61.8)	−0.99	317 (67.4)	−1.14	8 (72.7)	−1.30	171 (65.0)	−1.08	703 (62.7)	−1.01	17 (70.8)	−1.24	17 (63.0)	−1.02
Other psychiatric disorder	19 368 (24.2)	1658 (48.8)	−0.53	248 (46.5)	−0.48	440 (47.3)	−0.50	234 (49.8)	−0.55	5 (45.5)	−0.46	104 (39.5)	−0.34	591 (52.7)	−0.61	10 (41.7)	−0.38	16 (59.3)	−0.76
Mortality	6698 (8.4)	497 (14.6)	0.20	69 (12.9)	0.15	155 (16.7)	0.25	46 (9.8)	−0.05	2 (18.2)	0.29	37 (14.1)	0.18	180 (16.1)	0.24	2 (8.3)	0.00	4 (14.8)	0.20
Health care center type																			
Academic	7173 (8.9)	353 (10.4)	0.05	47 (8.8)	0.00	96 (10.3)	0.05	60 (12.8)	0.12	3 (27.3)	0.49	25 (9.5)	−0.02	111 (9.9)	−0.03	4 (16.7)	0.23	1 (3.7)	0.22
Children	245 (0.3)	2 (0.1)	0.06	1 (0.2)	0.02	0	0.08	0	0.08	0	0.08	0	0.08	1 (0.1)	0.05	0	0.08	0	0.08
Community health care	188 (0.2)	5 (0.1)	0.02	0	0.07	2 (0.2)	0.00	0	0.07	0	0.07	0	0.07	3 (0.3)	−0.01	0	0.07	0	0.07
Community hospital	506 (0.6)	27 (0.8)	−0.02	6 (1.1)	−0.05	7 (0.8)	−0.02	4 (0.9)	−0.03	0	0.11	2 (0.8)	−0.02	8 (0.7)	−0.01	0	0.11	0	0.11
Critical access	41 (0.1)	1 (0.03)	0.01	0	0.03	1 (0.1)	0.02	0	0.03	0	0.03	0	0.03	0	0.03	0	0.03	0	0.03
IDN	62 342 (77.8)	2586 (75.5)	0.04	405 (76.0)	0.04	708 (76.1)	0.04	342 (72.8)	0.12	7 (63.6)	0.31	202 (76.8)	0.02	866 (77.3)	0.01	18 (75.0)	0.07	24 (88.9)	−0.30
Regional hospital	9686 (12.1)	427 (12.6)	−0.01	74 (13.9)	−0.05	116 (12.5)	−0.01	64 (13.6)	−0.05	1 (9.1)	−0.10	34 (12.9)	−0.03	132 (11.8)	0.01	2 (8.3)	−0.12	2 (7.4)	−0.16
Region																			
Northeast	17 550 (21.9)	603 (17.7)	0.10	93 (17.4)	0.11	186 (20.0)	0.05	79 (16.8)	0.13	6 (54.5)	−0.71	41 (15.6)	0.16	185 (16.5)	0.14	6 (25.0)	−0.07	3 (11.1)	0.29
Midwest	6232 (7.8)	367 (10.8)	0.10	60 (11.3)	0.12	95 (10.2)	0.09	64 (13.6)	0.19	1 (9.1)	−0.05	25 (9.5)	−0.06	113 (10.1)	0.08	5 (20.8)	0.38	1 (3.7)	0.18
South	32 059 (40.0)	1522 (44.8)	−0.10	230 (43.2)	−0.06	428 (46.0)	−0.12	220 (46.8)	−0.14	3 (27.3)	0.27	118 (44.9)	−0.10	483 (43.1)	−0.06	10 (41.7)	−0.03	18 (66.7)	−0.56
West	24 340 (30.4)	909 (26.7)	0.08	150 (28.1)	0.05	221 (23.8)	0.15	107 (22.8)	0.17	1 (9.1)	−0.56	79 (30.0)	0.01	340 (30.3)	0.00	3 (12.5)	0.45	5 (18.5)	0.28

Abbreviations: COPD, chronic obstructive pulmonary disease; CVD, cerebrovascular disease; IDN, integrated delivery network; SMD, standardized mean difference; SSRI, selective serotonin reuptake inhibitor.

^a^ Unless otherwise indicated, data are expressed as number (%) of patients.

The mortality rate among SSRI-treated patients was 14.6% (497 of 3401) and among matched untreated control patients ranged from 16.3% (1107 of 6802) to 16.6% (1130 of 6802), with a reduction of 8% in the RR (0.92 [95% CI, 0.85-0.99]; adjusted *P* = .03, with any reduction in the RR of mortality significant for 10 of 10 iterations) (Table 3 and eTable 5 in the Supplement). The mortality rate among fluoxetine-treated patients was 9.8% (46 of 470) and among matched untreated control patients ranged from 13.3% (937 of 7050) to 13.4% (942 of 7050), with a reduction of 28% in the RR (0.72 [95% CI, 0.54-0.97]; adjusted *P* = .03, with any RR reduction statistically significant for 10 of 10 iterations) (Table 3 and eTable 6 in the Supplement). The mortality rate among fluoxetine- or fluvoxamine-treated patients was 10.0% (48 of 481) and among matched treated control patients ranged from 13.3% (956 of 7215) to 13.4% (964 of 7215), with a reduction of 26% in the RR (0.74 [95% CI, 0.55-0.99]; adjusted *P* = .04, with any reduction in RR statistically significant for 10 of 10 iterations) (Table 3 and eTable 7 in the Supplement). The mortality rate among patients treated with an SSRI other than fluoxetine or fluvoxamine was 15.4% (447 of 2898) and among matched untreated control patients ranged from 17.0% (1474 of 8694) to 17.3% (1501 of 8694), with a reduction of 8% in the RR (0.92 [95% CI, 0.84-1.00]; adjusted *P* = .06, with any reduction in RR statistically significant for 7 of 10 iterations) (Table 3 and eTable 8 in the Supplement).

**Table 3.** Mortality Rate for Treated Patients and Propensity Score–Matched Control Patients

SSRI	Treated patients		Control patients		RR (95% CI)	Adjusted *P* value^a^
	Mortality rate, %	No. died/No. treated	Mortality rate, %	No. died/total No.		
Any	14.6	497/3401	16.3	1107/6802	0.92 (0.85-0.99)	.03
Fluoxetine	9.8	46/470	13.3	937/7050	0.72 (0.54-0.97)	.03
Fluoxetine or fluvoxamine	10.0	48/481	13.3	956/7215	0.74 (0.55-0.99)	.04
Other (not fluoxetine or fluvoxamine)	15.4	447/2898	17.0	1474/8694	0.92 (0.84-1.00)	.06

Abbreviations: RR, relative risk; SSRI, selective serotonin reuptake inhibitor.

^a^ Benjamini-Hochberg adjusted *P* value from the iteration with the least significant result for each comparison.

For patients receiving any SSRI, the mean (SD) fluoxetine-equivalent dose was 30.2 (22.6) mg/d, with dose information available for 3008 of the 3401 patients (88.4%). For patients receiving fluoxetine specifically, the mean (SD) fluoxetine dose was 28.2 (16.9) mg/d, with dose information available for 404 of the 470 patients (86.0%). For patients receiving fluoxetine or fluvoxamine, the mean (SD) fluoxetine-equivalent dose was 29.0 (18.0) mg/d, with dose information available for 414 of the 481 patients (86.1%). For patients receiving an SSRI other than fluvoxamine, the mean (SD) fluoxetine-equivalent dose was 30.4 (23.1) mg/d, with dose information available for 2558 of the 2898 patients (88.3%).

## Discussion

We observed a small, statistically significant reduction of 8% in the RR of mortality among patients with COVID-19 prescribed SSRIs when compared with matched control patients. Our subgroup analysis found a statistically significant reduction of 28% in the RR of mortality for the patients treated with fluoxetine and 26% for the patients treated with fluoxetine or fluvoxamine. For the subgroup of patients treated with SSRIs other than fluoxetine or fluvoxamine, there was an 8% reduction in the RR of mortality, although this finding did not meet our significance threshold for every iteration performed. Although a previous investigation^2^ showed that fluvoxamine—an SSRI with properties similar to that of fluoxetine—can reduce severity of COVID-19 outcomes, the number of patients using fluvoxamine was too small in our EHR database to explore this medication independently or draw any robust conclusions.

A reported 13.2% of people 18 years or older used antidepressant medications in the US within the past 30 days,^27^ and SSRIs account for more than half of the antidepressants taken by patients,^9^ yet the proportion of patients receiving SSRIs in our analysis was just 4.1% (3401 of 83 584 patients). Some of this discrepancy may be explained by our narrower time frame (17 days around the time of a patient’s COVID-19 diagnosis), our patient population representing only those who are in an urgent care or higher-acuity clinical setting, or possible underreporting of medications in the Cerner Real World Data EHR. Subsequently, there may be a potential underestimation of the strength of the associations with SSRIs and COVID-19.

Several mechanisms by which SSRIs can reduce the severity of COVID-19 symptoms have been proposed in the literature. The severe respiratory illness of COVID-19 is primarily triggered by an intense proinflammatory host response.^7^ Selective serotonin reuptake inhibitors may benefit patients with COVID-19 owing to the link between serotonin and the immune system.^28,29,30^ More specifically, severe outcomes of COVID-19 have been associated with several proinflammatory cytokines, including interleukin 6, whose increased levels contribute to the cytokine storm.^7^ Various studies have indicated that SSRIs and specifically fluoxetine can decrease levels of these cytokines and interleukin 6 signaling activity.^31,32^ Some SSRIs, such as fluoxetine and fluvoxamine, may modulate the sigma-1 receptor-IRE1 pathway, thereby reducing damaging aspects of the inflammatory response.^33^ Another potential explanation could be related to their inhibiting effect on the acid sphingomyelinase/ceramide system,^1^ whose activation may play an important role in SARS-CoV-2 infection because it leads to the formation of ceramide-enriched membrane domains that facilitate viral entry and infection by clustering angiotensin-converting enzyme 2, the cellular receptor of SARS-CoV-2.^13,14^ Metabolic markers of ceramide metabolism have also been associated with respiratory severity and inflammation in patients hospitalized for COVID-19.^15^ Finally, some evidence suggests that SSRIs and especially fluoxetine could have antiviral effects.^4,34,35^

### Limitations

One of the main limitations of our study is that its retrospective nature only allowed us to identify an association between SSRI treatment and COVID-19 mortality, but not causal effects. Moreover, although records are available from 2015 and beyond for some individuals in this EHR database, this was not the case for all individuals; therefore, pertinent information such as previous medication use and comorbidities for certain individuals could be incomplete in this database. Our study included medication orders for SSRIs with order statuses that ensured that the medications were administered to the individuals—an advantage compared with outpatient prescriptions, where there can be uncertainty as to whether an individual actually takes the medication or merely fills the prescription. However, we did not consider the administration of specific SSRIs beyond fluoxetine and at various doses, which could have differing pleiotropic effects in lieu of their designed effect. Nonetheless, the fact that any mitigating association with COVID-19–related mortality was found is intriguing. We considered several demographic characteristics and comorbidities known to be associated with COVID-19 outcomes, but unaccounted confounding variables could alter this association. Although we examined medical conditions associated with severe COVID-19 as independent variables in our model, one could consider the total number of medical diseases (categorized in a few classes) in addition to these variables. In the absence of collinearity, this additional variable may improve the adjustment. Over time, it is feasible that the difference in mortality or morbidity attributed to SSRI treatment compared with control patients not using SSRIs might lessen as the overall mortality and morbidity rates for COVID-19 decrease because of general improvement in care with other anti-inflammatory regimens or specific treatments.

## Conclusions

In this cohort study, the RR of mortality was reduced 8% among patients prescribed any SSRI and 28% among those prescribed fluoxetine. These findings suggest that SSRIs, if proven effective, could be a therapeutic option to reduce mortality among patients with COVID-19. Further research and large, randomized clinical trials are needed to elucidate the effect of SSRIs generally, or more specifically of fluoxetine and fluvoxamine, on the severity of COVID-19 outcomes.

## Notes

Supplement. **eFigure 1.** Propensity Scores (PS) and Standardized Mean Differences (SMD) of Covariates for Comparison of SSRI-Treated Patients and Matched Control Patients **eFigure 2.** Propensity Scores (PS) and Standardized Mean Differences (SMD) of Covariates for Comparison of Fluoxetine-Treated Patients and Matched Control Patients **eFigure 3.** Propensity Scores (PS) and Standardized Mean Differences (SMD) of Covariates for Comparison of Fluoxetine- or Fluvoxamine-Treated Patients and Matched Control Patients **eFigure 4.** Propensity Scores (PS) and Standardized Mean Differences (SMD) of Covariates for Comparison of Other (Not Fluoxetine or Fluvoxamine) SSRI-Treated Patients and Matched Control Patients **eTable 1.** Standardized Mean Differences (SMD) of Covariates Between SSRI-Treated and Control Groups Before and After Matching **eTable 2.** Standardized Mean Differences (SMD) of Covariates Between Fluoxetine-Treated and Control Groups Before and After Matching **eTable 3.** Standardized Mean Differences (SMD) of Covariates Between Fluoxetine- or Fluvoxamine-Treated and Control Groups Before and After Matching **eTable 4.** Standardized Mean Differences (SMD) of Covariates Between Other (Not Fluoxetine or Fluvoxamine) SSRI-Treated and Control Groups Before and After Matching **eTable 5.** Propensity Score (PS) Matching by Demographics, Encounter Type at the Time of the First Recorded COVID-19 Diagnosis, COVID-19 Comorbidities, and Prescription Indications for SSRI-Exposed Patients and Unexposed Control Patients **eTable 6.** Propensity Score (PS) Matching by Demographics, Encounter Type at the Time of the First Recorded COVID-19 Diagnosis, COVID-19 Comorbidities, and Prescription Indications for Fluoxetine-Exposed Patients and Unexposed Control Patients **eTable 7.** Propensity Score (PS) Matching by Demographics, Encounter Type at the Time of the First Recorded COVID-19 Diagnosis, COVID-19 Comorbidities, and Prescription Indications for Fluoxetine- or Fluvoxamine-Exposed Patients and Unexposed Control Patients **eTable 8.** Propensity Score (PS) Matching by Demographics, Encounter Type at the Time of the First Recorded COVID-19 Diagnosis, COVID-19 Comorbidities, and Prescription Indications for Other (Not Fluoxetine or Fluvoxamine) SSRI-Exposed Patients and Unexposed Control Patients
