# Supplementary material for: Mortality Risk Among Patients With COVID-19 Prescribed Selective Serotonin Reuptake Inhibitor Antidepressants
Source: JAMA Netw Open. 2021 Nov 15;4(11):e2133090. doi: 10.1001/jamanetworkopen.2021.33090 (PMC8593759; doi:10.1001/jamanetworkopen.2021.33090)
Supplement: Supplement. — eFigure 1. Propensity Scores (PS) and Standardized Mean Differences (SMD) of Covariates for Comparison of SSRI-Treated Patients and Matched Control Patients eFigure 2. Propensity Scores (PS) and Standardized Mean Differences (SMD) of Covariates for Comparison of Fluoxetine-Treated Patients and Matched Control Patients eFigure 3. Propensity Scores (PS) and Standardized Mean Differences (SMD) of Covariates for Comparison of Fluoxetine- or Fluvoxamine-Treated Patients and Matched Control Patients eFigure 4. Propensity Scores (PS) and Standardized Mean Differences (SMD) of Covariates for Comparison of Other (Not Fluoxetine or Fluvoxamine) SSRI-Treated Patients and Matched Control Patients eTable 1. Standardized Mean Differences (SMD) of Covariates Between SSRI-Treated and Control Groups Before and After Matching eTable 2. Standardized Mean Differences (SMD) of Covariates Between Fluoxetine-Treated and Control Groups Before and After Matching eTable 3. Standardized Mean Differences (SMD) of Covariates Between Fluoxetine- or Fluvoxamine-Treated and Control Groups Before and After Matching eTable 4. Standardized Mean Differences (SMD) of Covariates Between Other (Not Fluoxetine or Fluvoxamine) SSRI-Treated and Control Groups Before and After Matching eTable 5. Propensity Score (PS) Matching by Demographics, Encounter Type at the Time of the First Recorded COVID-19 Diagnosis, COVID-19 Comorbidities, and Prescription Indications for SSRI-Exposed Patients and Unexposed Control Patients eTable 6. Propensity Score (PS) Matching by Demographics, Encounter Type at the Time of the First Recorded COVID-19 Diagnosis, COVID-19 Comorbidities, and Prescription Indications for Fluoxetine-Exposed Patients and Unexposed Control Patients eTable 7. Propensity Score (PS) Matching by Demographics, Encounter Type at the Time of the First Recorded COVID-19 Diagnosis, COVID-19 Comorbidities, and Prescription Indications for Fluoxetine- or Fluvoxamine-Exposed Patients and Unexposed Control Patien [file jamanetwopen-e2133090-s001.pdf]

Supplementary Online Content

Oskotsky T, Marić I, Tang A, et al. Mortality risk among patients with COVID-19 prescribed selective serotonin reuptake inhibitor antidepressants. *JAMA Netw Open*. 2021;4(11):e2133090. doi:10.1001/jamanetworkopen.2021.33090

eFigure 1. Propensity Scores (PS) and Standardized Mean Differences (SMD) of Covariates for Comparison of SSRI-Treated Patients and Matched Control Patients
eFigure 2. Propensity Scores (PS) and Standardized Mean Differences (SMD) of Covariates for Comparison of Fluoxetine-Treated Patients and Matched Control Patients
eFigure 3. Propensity Scores (PS) and Standardized Mean Differences (SMD) of Covariates for Comparison of Fluoxetine- or Fluvoxamine-Treated Patients and Matched Control Patients
eFigure 4. Propensity Scores (PS) and Standardized Mean Differences (SMD) of Covariates for Comparison of Other (Not Fluoxetine or Fluvoxamine) SSRI-Treated Patients and Matched Control Patients
eTable 1. Standardized Mean Differences (SMD) of Covariates Between SSRI-Treated and Control Groups Before and After Matching
eTable 2. Standardized Mean Differences (SMD) of Covariates Between Fluoxetine-Treated and Control Groups Before and After Matching
eTable 3. Standardized Mean Differences (SMD) of Covariates Between Fluoxetine- or Fluvoxamine-Treated and Control Groups Before and After Matching
eTable 4. Standardized Mean Differences (SMD) of Covariates Between Other (Not Fluoxetine or Fluvoxamine) SSRI-Treated and Control Groups Before and After Matching
eTable 5. Propensity Score (PS) Matching by Demographics, Encounter Type at the Time of the First Recorded COVID-19 Diagnosis, COVID-19 Comorbidities, and Prescription Indications for SSRI-Exposed Patients and Unexposed Control Patients
eTable 6. Propensity Score (PS) Matching by Demographics, Encounter Type at the Time of the First Recorded COVID-19 Diagnosis, COVID-19 Comorbidities, and Prescription Indications for Fluoxetine-Exposed Patients and Unexposed Control Patients
eTable 7. Propensity Score (PS) Matching by Demographics, Encounter Type at the Time of the First Recorded COVID-19 Diagnosis, COVID-19 Comorbidities, and Prescription Indications for Fluoxetine- or Fluvoxamine-Exposed Patients and Unexposed Control Patients
eTable 8. Propensity Score (PS) Matching by Demographics, Encounter Type at the Time of the First Recorded COVID-19 Diagnosis, COVID-19 Comorbidities, and Prescription Indications for Other (Not Fluoxetine or Fluvoxamine) SSRI-Exposed Patients and Unexposed Control Patients

This supplementary material has been provided by the authors to give readers additional information about their work.

© 2021 Oskotsky T et al. *JAMA Network Open*.

eFigure 1. Propensity Scores (PS) and Standardized Mean Differences (SMD) of Covariates for Comparison of SSRI-Treated Patients and Matched Control Patients

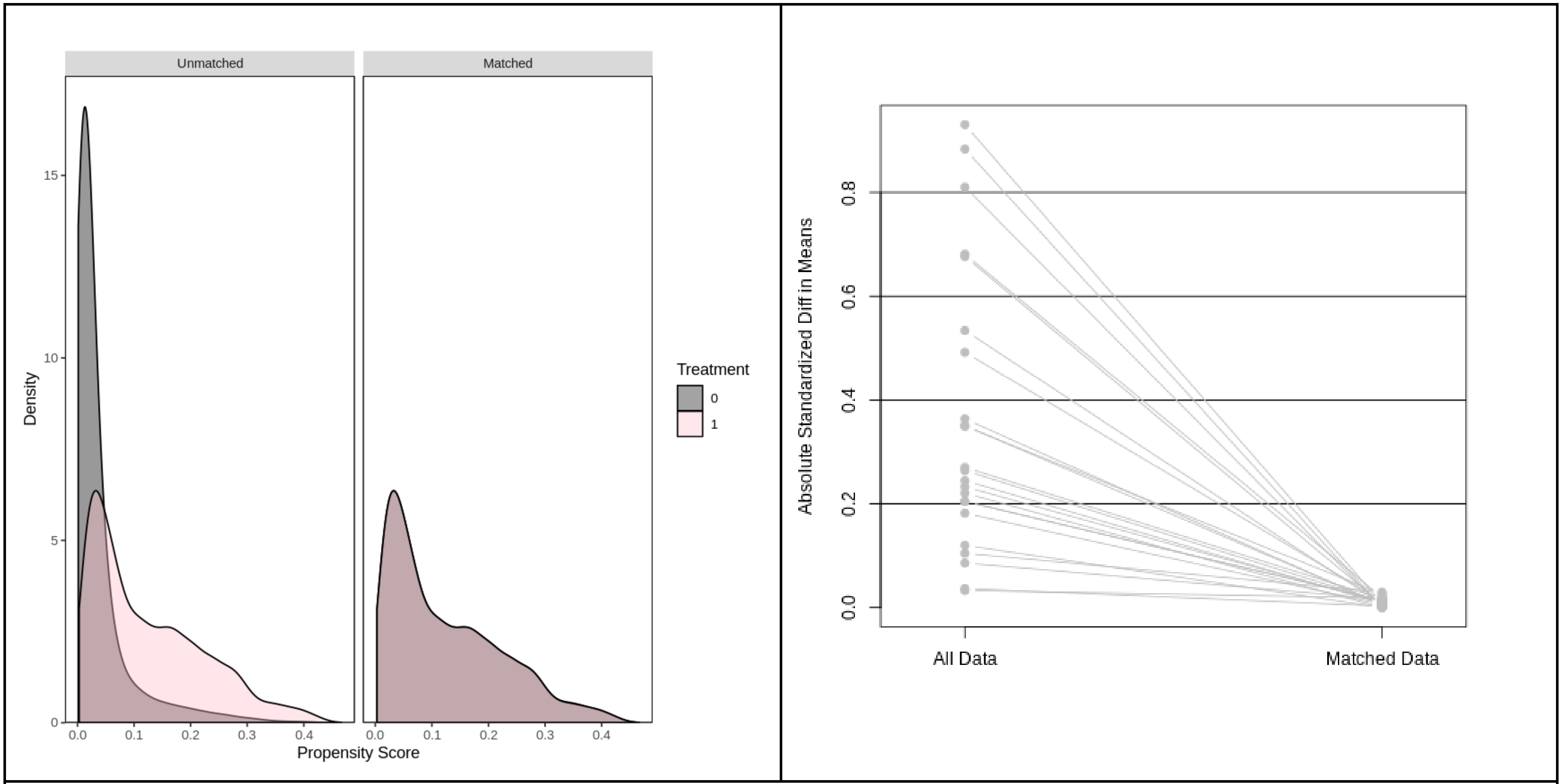

(a) Density plots of the distribution of propensity scores for the SSRI-treated and control groups before and after matching. **(b)** Absolute standardized differences in means between SSRI-treated and control groups for all data (before matching) and matched data.

© 2021 Oskotsky T et al. *JAMA Network Open*.

eFigure 2. Propensity Scores (PS) and Standardized Mean Differences (SMD) of Covariates for Comparison of Fluoxetine-Treated Patients and Matched Control Patients

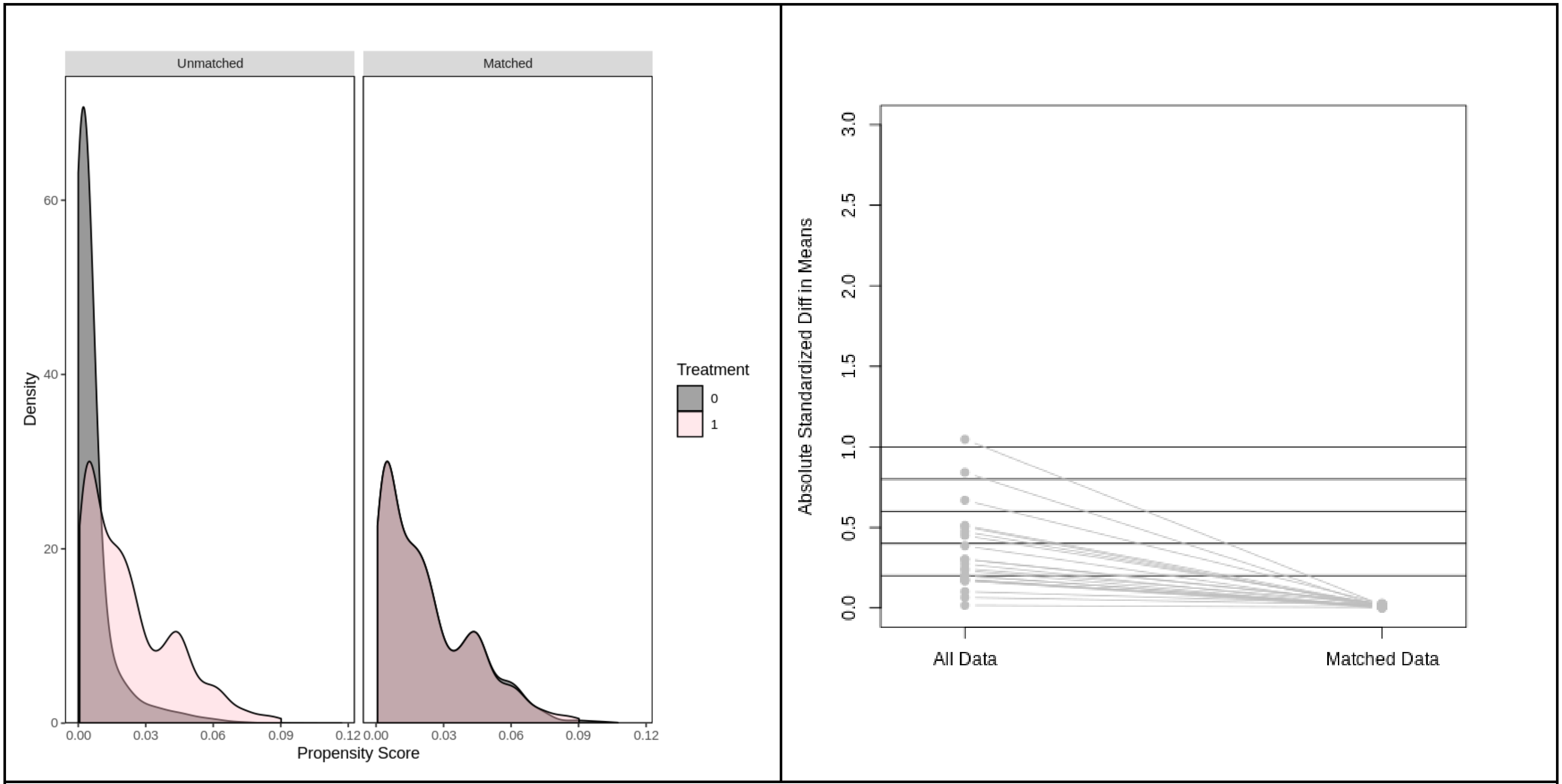

(a) Density plots of the distribution of propensity scores for the fluoxetine-treated and control groups before and after matching. **(b)** Absolute standardized differences in means between fluoxetine-treated and control groups for all data (before matching) and matched data.

© 2021 Oskotsky T et al. *JAMA Network Open*.

eFigure 3. Propensity Scores (PS) and Standardized Mean Differences (SMD) of Covariates for Comparison of Fluoxetine- or Fluvoxamine-Treated Patients and Matched Control Patients

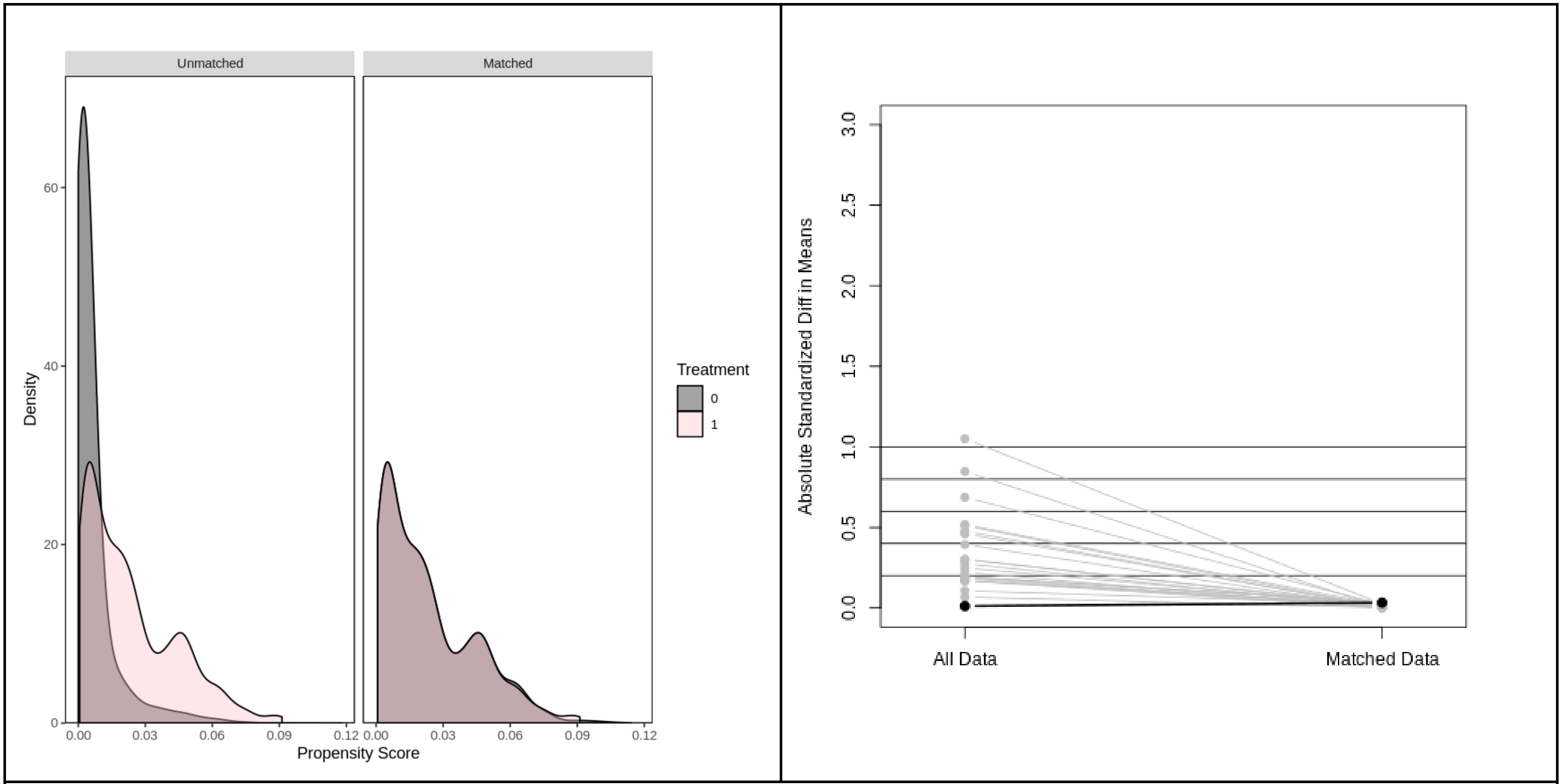

(a) Density plots of the distribution of propensity scores for the fluoxetine- or fluvoxamine-treated and control groups before and after matching. **(b)** Absolute standardized differences in means between fluoxetine- or fluvoxamine-treated and control groups for all data (before matching) and matched data.

© 2021 Oskotsky T et al. *JAMA Network Open*.

eFigure 4. Propensity Scores (PS) and Standardized Mean Differences (SMD) of Covariates for Comparison of Other (Not Fluoxetine or Fluvoxamine) SSRI-Treated Patients and Matched Control Patients

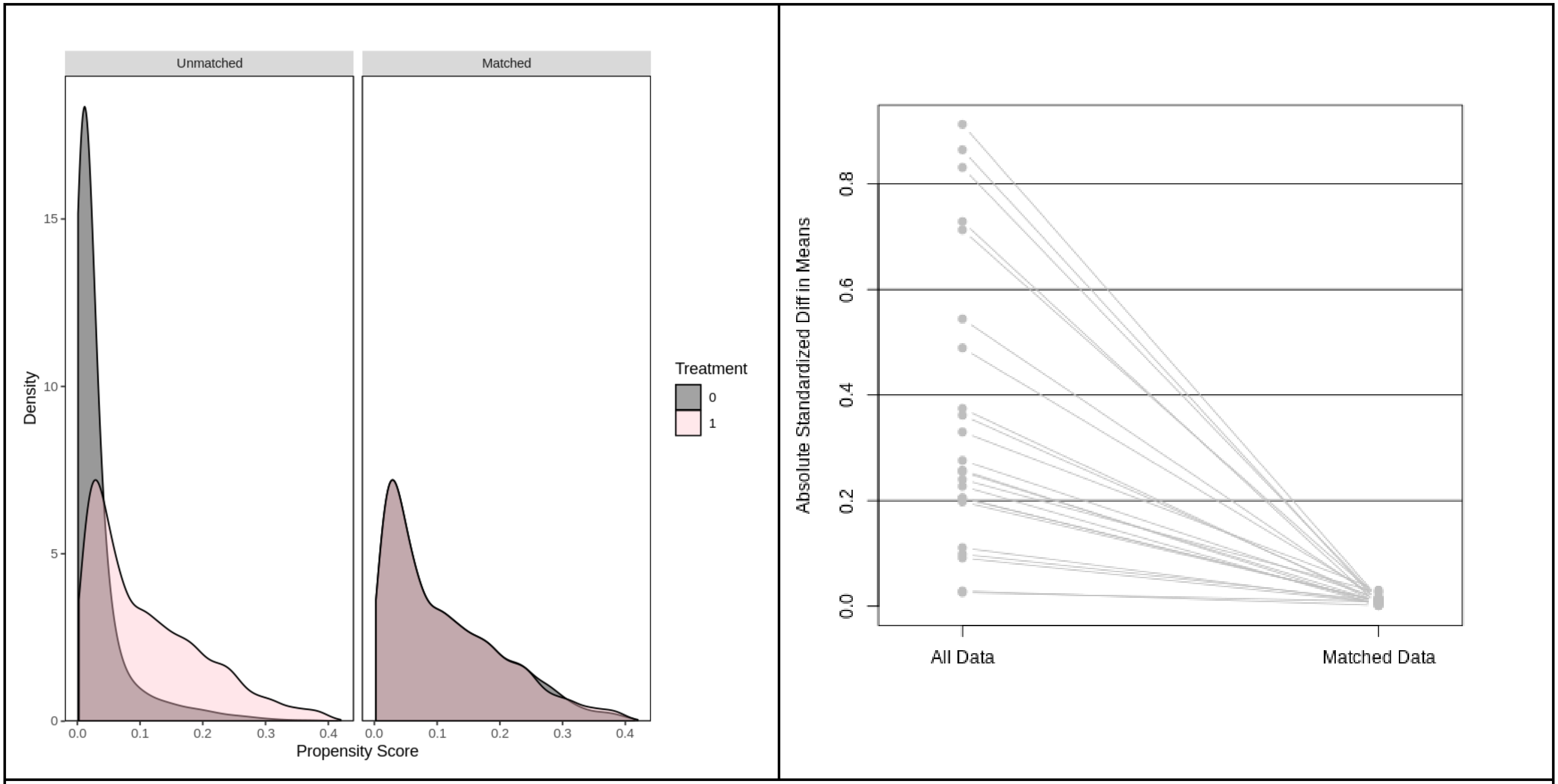

(a) Density plots of the distribution of propensity scores for the other SSRI-treated and control groups before and after matching. **(b)** Absolute standardized differences in means between other SSRI-treated and control groups for all data (before matching) and matched data.

© 2021 Oskotsky T et al. *JAMA Network Open*.

eTable 1. Standardized Mean Differences (SMD) of Covariates Between SSRI-Treated and Control Groups Before and After Matching

	Before Matching			After Matching		
	Means (SD) Treated	Means (SD) Control	SMD	Means (SD) Treated	Means (SD) Control	SMD
Age	63.83 (18.07)	51.61 (19.18)	0.66	63.83 (18.07)	63.71 (17.87)	0.01
Female	0.60 (0.49)	0.50 (0.50)	0.20	0.60 (0.49)	0.60 (0.49)	0.01
Male	0.40 (0.49)	0.50 (0.50)	-0.20	0.40 (0.49)	0.40 (0.49)	0.01
American Indian or Alaska Native	0.01 (0.11)	0.02 (0.14)	-0.07	0.01 (0.11)	0.01 (0.11)	0.00
Asian or Pacific Islander	0.01 (0.11)	0.02 (0.16)	-0.10	0.01 (0.11)	0.01 (0.11)	<0.001
Black or African American	0.14 (0.35)	0.20 (0.40)	-0.17	0.14 (0.35)	0.14 (0.35)	0.01
Mixed Racial Group	0.00 (0.05)	0.00 (0.06)	0.03	0.00 (0.05)	0.00 (0.05)	0.00
Other Racial Group	0.07 (0.25)	0.14 (0.34)	-0.23	0.07 (0.25)	0.06 (0.24)	0.02
White	0.77 (0.42)	0.61 (0.49)	0.34	0.77 (0.42)	0.77 (0.42)	0.01
Hispanic or Latino	0.29 (0.45)	0.44 (0.50)	-0.33	0.29 (0.45)	0.27 (0.45)	0.03
Not Hispanic or Latino	0.71 (0.45)	0.56 (0.50)	0.33	0.71 (0.45)	0.73 (0.45)	-0.03
Admitted for Observation	0.06 (0.25)	0.04 (0.19)	0.12	0.06 (0.25)	0.06 (0.23)	0.03
Emergency	0.19 (0.39)	0.51 (0.50)	-0.71	0.19 (0.39)	0.19 (0.39)	<0.001
Inpatient	0.74 (0.44)	0.44 (0.50)	0.64	0.74 (0.44)	0.75 (0.43)	-0.02
Urgent care encounter	0.01 (0.07)	0.01 (0.11)	0.08	0.01 (0.07)	0.00 (0.06)	0.03
Obese	0.39 (0.49)	0.37 (0.48)	0.03	0.39 (0.49)	0.40 (0.49)	-0.02
Cancer	0.07 (0.26)	0.05 (0.22)	0.09	0.07 (0.26)	0.07 (0.26)	0.02
Cerebrovascular Disease	0.16 (0.36)	0.07 (0.26)	0.27	0.16 (0.36)	0.15 (0.36)	0.01
Chronic Kidney Disease (CKD)	0.21 (0.41)	0.12 (0.33)	0.24	0.21 (0.41)	0.21 (0.41)	<0.001
Chronic Obstructive Pulmonary Disease (COPD)	0.18 (0.38)	0.08 (0.26)	0.31	0.18 (0.38)	0.17 (0.38)	0.01
Diabetes	0.43 (0.49)	0.30 (0.46)	0.25	0.43 (0.49)	0.43 (0.50)	0.01
Heart Diseases	0.36 (0.48)	0.19 (0.40)	0.38	0.36 (0.48)	0.37 (0.48)	-0.01
Hypertension	0.71 (0.46)	0.46 (0.50)	0.51	0.71 (0.46)	0.71 (0.45)	0.01
Mood or Anxiety Disorder	0.63 (0.48)	0.18 (0.39)	1.03	0.63 (0.48)	0.63 (0.48)	0.01
Other Psychiatric Disorder (Not Mood or Anxiety)	0.49 (0.50)	0.24 (0.43)	0.53	0.49 (0.50)	0.48 (0.50)	0.01

© 2021 Oskotsky T et al. *JAMA Network Open*.

eTable 2. Standardized Mean Differences (SMD) of Covariates Between Fluoxetine-Treated and Control Groups Before and After Matching

	Before Matching			After Matching		
	Means (SD) Treated	Means (SD) Control	SMD	Means (SD) Treated	Means (SD) Control	SMD
Age	58.59 (18.09)	51.61 (19.18)	0.38	58.59 (18.09)	58.50 (18.86)	0.01
Female	0.60 (0.49)	0.50 (0.50)	0.20	0.60 (0.49)	0.60 (0.49)	0.01
Male	0.40 (0.49)	0.50 (0.50)	-0.20	0.40 (0.49)	0.40 (0.49)	0.01
American Indian or Alaska Native	0.01 (0.09)	0.02 (0.14)	-0.10	0.01 (0.09)	0.01 (0.10)	0.01
Asian or Pacific Islander	0.01 (0.09)	0.02 (0.16)	-0.13	0.01 (0.09)	0.01 (0.10)	0.01
Black or African American	0.17 (0.37)	0.20 (0.40)	-0.10	0.17 (0.37)	0.17 (0.38)	0.02
Mixed Racial Group	0.00 (0.00)	0.00 (0.06)	0.09	0.00 (0.00)	0.00 (0.00)	<0.001
Other Racial Group	0.07 (0.26)	0.14 (0.34)	-0.20	0.07 (0.26)	0.07 (0.26)	<0.001
White	0.74 (0.44)	0.61 (0.49)	0.28	0.74 (0.44)	0.73 (0.44)	0.02
Hispanic or Latino	0.25 (0.43)	0.44 (0.50)	-0.42	0.25 (0.43)	0.24 (0.43)	0.02
Not Hispanic or Latino	0.75 (0.43)	0.56 (0.50)	0.42	0.75 (0.43)	0.76 (0.43)	-0.02
Admitted for Observation	0.09 (0.29)	0.04 (0.19)	0.23	0.09 (0.29)	0.08 (0.28)	0.03
Emergency	0.23 (0.42)	0.51 (0.50)	-0.61	0.23 (0.42)	0.24 (0.43)	-0.03
Inpatient	0.68 (0.47)	0.44 (0.50)	0.49	0.68 (0.47)	0.68 (0.47)	0.00
Urgent care encounter	0.00 (0.05)	0.01 (0.11)	-0.13	0.00 (0.05)	0.00 (0.03)	0.03
Obese	0.41 (0.49)	0.37 (0.48)	0.07	0.41 (0.49)	0.42 (0.49)	-0.02
Cancer	0.05 (0.22)	0.05 (0.22)	0.02	0.05 (0.22)	0.05 (0.22)	0.00
Cerebrovascular Disease	0.13 (0.34)	0.07 (0.26)	0.20	0.13 (0.34)	0.13 (0.34)	0.01
Chronic Kidney Disease (CKD)	0.19 (0.39)	0.12 (0.33)	0.18	0.19 (0.39)	0.19 (0.39)	<0.001
Chronic Obstructive Pulmonary Disease (COPD)	0.20 (0.40)	0.08 (0.26)	0.36	0.20 (0.40)	0.18 (0.39)	0.03
Diabetes	0.39 (0.49)	0.30 (0.46)	0.17	0.39 (0.49)	0.39 (0.49)	0.02
Heart Diseases	0.32 (0.47)	0.19 (0.40)	0.30	0.32 (0.47)	0.32 (0.47)	<0.001
Hypertension	0.69 (0.46)	0.46 (0.50)	0.46	0.69 (0.46)	0.69 (0.46)	0.01
Mood or Anxiety Disorder	0.67 (0.47)	0.18 (0.39)	1.14	0.67 (0.47)	0.68 (0.47)	-0.02

© 2021 Oskotsky T et al. *JAMA Network Open*.

Other Psychiatric Disorder (Not Mood or Anxiety)	0.50 (0.50)	0.24 (0.43)	0.55	0.50 (0.50)	0.49 (0.50)	0.02

© 2021 Oskotsky T et al. *JAMA Network Open*.

eTable 3. Standardized Mean Differences (SMD) of Covariates Between Fluoxetine- or Fluvoxamine-Treated and Control Groups Before and After Matching

	Before Matching			After Matching		
	Means (SD) Treated	Means (SD) Control	SMD	Means (SD) Treated	Means (SD) Control	SMD
Age	58.69 (17.96)	51.61 (19.18)	0.38	58.69 (17.96)	58.63 (19.02)	0.00
Female	0.59 (0.49)	0.50 (0.50)	0.19	0.59 (0.49)	0.61 (0.49)	-0.04
Male	0.41 (0.49)	0.50 (0.50)	-0.19	0.41 (0.49)	0.39 (0.49)	0.04
American Indian or Alaska Native	0.01 (0.09)	0.02 (0.14)	-0.11	0.01 (0.09)	0.01 (0.09)	0.00
Asian or Pacific Islander	0.01 (0.09)	0.02 (0.16)	-0.13	0.01 (0.09)	0.01 (0.09)	0.00
Black or African American	0.16 (0.37)	0.20 (0.40)	-0.10	0.16 (0.37)	0.17 (0.38)	-0.03
Mixed Racial Group	0.00 (0.00)	0.00 (0.06)	0.09	0.00 (0.00)	0.00 (0.00)	<0.001
Other Racial Group	0.08 (0.27)	0.14 (0.34)	-0.19	0.08 (0.27)	0.08 (0.27)	0.00
White	0.74 (0.44)	0.61 (0.49)	0.28	0.74 (0.44)	0.73 (0.44)	0.02
Hispanic or Latino	0.25 (0.43)	0.44 (0.50)	-0.43	0.25 (0.43)	0.24 (0.43)	0.01
Not Hispanic or Latino	0.75 (0.43)	0.56 (0.50)	0.43	0.75 (0.43)	0.76 (0.43)	-0.01
Admitted for Observation	0.09 (0.29)	0.04 (0.19)	0.23	0.09 (0.29)	0.08 (0.27)	0.06
Emergency	0.22 (0.42)	0.51 (0.50)	-0.62	0.22 (0.42)	0.23 (0.42)	-0.02
Inpatient	0.68 (0.47)	0.44 (0.50)	0.50	0.68 (0.47)	0.69 (0.46)	-0.02
Urgent care encounter	0.00 (0.05)	0.01 (0.11)	-0.13	0.00 (0.05)	0.00 (0.04)	0.02
Obese	0.41 (0.49)	0.37 (0.48)	0.07	0.41 (0.49)	0.41 (0.49)	0.00
Cancer	0.05 (0.22)	0.05 (0.22)	0.01	0.05 (0.22)	0.06 (0.23)	-0.03
Cerebrovascular Disease	0.14 (0.34)	0.07 (0.26)	0.21	0.14 (0.34)	0.14 (0.35)	0.01
Chronic Kidney Disease (CKD)	0.19 (0.39)	0.12 (0.33)	0.18	0.19 (0.39)	0.20 (0.40)	-0.02
Chronic Obstructive Pulmonary Disease (COPD)	0.20 (0.40)	0.08 (0.26)	0.36	0.20 (0.40)	0.19 (0.39)	0.01
Diabetes	0.39 (0.49)	0.30 (0.46)	0.17	0.39 (0.49)	0.39 (0.49)	0.00
Heart Diseases	0.32 (0.47)	0.19 (0.40)	0.30	0.32 (0.47)	0.32 (0.47)	0.00
Hypertension	0.69 (0.46)	0.46 (0.50)	0.46	0.69 (0.46)	0.69 (0.46)	0.00
Mood or Anxiety Disorder	0.68 (0.47)	0.18 (0.39)	1.15	0.68 (0.47)	0.69 (0.46)	-0.03

© 2021 Oskotsky T et al. *JAMA Network Open*.

Other Psychiatric Disorder (Not Mood or Anxiety)	0.50 (0.50)	0.24 (0.43)	0.55	0.50 (0.50)	0.49 (0.50)	0.01

© 2021 Oskotsky T et al. *JAMA Network Open*.

eTable 4. Standardized Mean Differences (SMD) of Covariates Between Other (Not Fluoxetine or Fluvoxamine) SSRI-Treated and Control Groups Before and After Matching

	Before Matching			After Matching		
	**Means (SD) Treated**	**Means (SD) Control**	**SMD**	**Means (SD) Treated**	**Means (SD) Control**	**SMD**
Age	64.69 (17.95)	51.61 (19.18)	0.70	64.69 (17.95)	64.73 (17.68)	0.00
Female	0.60 (0.49)	0.50 (0.50)	0.20	0.60 (0.49)	0.59 (0.49)	0.01
Male	0.40 (0.49)	0.50 (0.50)	-0.20	0.40 (0.49)	0.41 (0.49)	-0.01
American Indian or Alaska Native	0.01 (0.12)	0.02 (0.14)	-0.06	0.01 (0.12)	0.01 (0.10)	0.02
Asian or Pacific Islander	0.01 (0.11)	0.02 (0.16)	-0.09	0.01 (0.11)	0.01 (0.11)	0.01
Black or African American	0.14 (0.34)	0.20 (0.40)	-0.18	0.14 (0.34)	0.14 (0.35)	0.01
Mixed Racial Group	0.00 (0.05)	0.00 (0.06)	0.02	0.00 (0.05)	0.00 (0.05)	0.01
Other Racial Group	0.07 (0.25)	0.14 (0.34)	-0.23	0.07 (0.25)	0.06 (0.24)	0.02
White	0.77 (0.42)	0.61 (0.49)	0.35	0.77 (0.42)	0.77 (0.42)	0.01
Hispanic or Latino	0.29 (0.46)	0.44 (0.50)	-0.32	0.29 (0.46)	0.28 (0.45)	0.03
Not Hispanic or Latino	0.71 (0.46)	0.56 (0.50)	0.32	0.71 (0.46)	0.72 (0.45)	-0.03
Admitted for Observation	0.06 (0.24)	0.04 (0.19)	0.10	0.06 (0.24)	0.06 (0.23)	0.00
Emergency	0.19 (0.39)	0.51 (0.50)	-0.72	0.19 (0.39)	0.18 (0.38)	0.01
Inpatient	0.75 (0.43)	0.44 (0.50)	0.66	0.75 (0.43)	0.76 (0.43)	-0.02
Urgent care encounter	0.01 (0.08)	0.01 (0.11)	0.07	0.01 (0.08)	0.01 (0.08)	0.01
Obese	0.39 (0.49)	0.37 (0.48)	0.03	0.39 (0.49)	0.39 (0.49)	0.00
Cancer	0.08 (0.27)	0.05 (0.22)	0.11	0.08 (0.27)	0.08 (0.27)	0.01
Cerebrovascular Disease	0.16 (0.37)	0.07 (0.26)	0.28	0.16 (0.37)	0.15 (0.36)	0.03
Chronic Kidney Disease (CKD)	0.22 (0.41)	0.12 (0.33)	0.25	0.22 (0.41)	0.22 (0.41)	0.00
Chronic Obstructive Pulmonary Disease (COPD)	0.17 (0.38)	0.08 (0.26)	0.30	0.17 (0.38)	0.17 (0.37)	0.01
Diabetes	0.43 (0.50)	0.30 (0.46)	0.27	0.43 (0.50)	0.43 (0.50)	0.00
Heart Diseases	0.37 (0.48)	0.19 (0.40)	0.40	0.37 (0.48)	0.37 (0.48)	0.01
Hypertension	0.71 (0.45)	0.46 (0.50)	0.52	0.71 (0.45)	0.72 (0.45)	-0.01
Mood or Anxiety Disorder	0.63 (0.48)	0.18 (0.39)	1.01	0.63 (0.48)	0.62 (0.49)	0.02

© 2021 Oskotsky T et al. *JAMA Network Open*.

Other Psychiatric Disorder (Not Mood or Anxiety)	0.49 (0.50)	0.24 (0.43)	0.53	0.49 (0.50)	0.48 (0.50)	0.02

© 2021 Oskotsky T et al. *JAMA Network Open*.

eTable 5. Propensity Score (PS) Matching by Demographics, Encounter Type at the Time of the First Recorded COVID-19 Diagnosis, COVID-19 Comorbidities, and Prescription Indications for SSRI-Exposed Patients and Unexposed Control Patients

Iteration	Exposed to SSRI, died, n (%), (n total = 3401)	Matched controls, died, n (%), (n total = 6802)	Difference in mortality rate (%)	RR [95% CI]	p-value (adjusted)
1	497 (14.6)	1114 (16.4)	-1.8	0.91 [0.84–0.99]	0.027
2	497 (14.6)	1110 (16.3)	-1.7	0.92 [0.85–0.99]	0.029
3	497 (14.6)	1126 (16.6)	-1.9	0.91 [0.84–0.98]	0.022
4	497 (14.6)	1123 (16.5)	-1.9	0.91 [0.84–0.98]	0.022
5	497 (14.6)	1123 (16.5)	-1.9	0.91 [0.84–0.98]	0.022
6	497 (14.6)	1124 (16.5)	-1.9	0.91 [0.84–0.98]	0.022
7	497 (14.6)	1107 (16.3)	-1.7	0.92 [0.85–0.99]	0.030
8	497 (14.6)	1130 (16.6)	-2.0	0.90 [0.83–0.98]	0.022
9	497 (14.6)	1121 (16.5)	-1.9	0.91 [0.84–0.98]	0.022
10	497 (14.6)	1125 (16.5)	-1.9	0.91 [0.84–0.98]	0.022

© 2021 Oskotsky T et al. *JAMA Network Open*.

eTable 6. Propensity Score (PS) Matching by Demographics, Encounter Type at the Time of the First Recorded COVID-19 Diagnosis, COVID-19 Comorbidities, and Prescription Indications for Fluoxetine-Exposed Patients and Unexposed Control Patients

Iteration	Exposed to fluoxetine, Died, n (%), (n total = 470)	Matched controls, Died, n (%), (n total = 7050)	Difference in mortality rate (%)	RR [95% CI]	p-value (adjusted)
1	46 (9.8)	940 (13.3)	-3.5	0.72 [0.53–0.97]	0.029
2	46 (9.8)	940 (13.3)	-3.5	0.72 [0.53–0.97]	0.029
3	46 (9.8)	941 (13.3)	-3.6	0.72 [0.53–0.97]	0.029
4	46 (9.8)	941 (13.3)	-3.6	0.72 [0.53–0.97]	0.029
5	46 (9.8)	937 (13.3)	-3.5	0.72 [0.54–0.97]	0.029
6	46 (9.8)	942 (13.4)	-3.6	0.72 [0.53–0.97]	0.029
7	46 (9.8)	942 (13.4)	-3.6	0.72 [0.53–0.97]	0.029
8	46 (9.8)	942 (13.4)	-3.6	0.72 [0.53–0.97]	0.029
9	46 (9.8)	937 (13.3)	-3.5	0.72 [0.54–0.97]	0.029
10	46 (9.8)	939 (13.3)	-3.5	0.72 [0.54–0.97]	0.029

© 2021 Oskotsky T et al. *JAMA Network Open*.

eTable 7. Propensity Score (PS) Matching by Demographics, Encounter Type at the Time of the First Recorded COVID-19 Diagnosis, COVID-19 Comorbidities, and Prescription Indications for Fluoxetine- or Fluvoxamine-Exposed Patients and Unexposed Control Patients

Iteration	Exposed to fluoxetine or fluvoxamine, died, n (%), (n total = 481)	Matched controls, died, n (%), (n total = 7215)	Difference in mortality rate (%)	RR [95% CI]	p-value (adjusted)
1	48 (10.0)	960 (13.3)	-3.3	0.74 [0.55–0.98]	0.042
2	48 (10.0)	958 (13.3)	-3.3	0.74 [0.55–0.99]	0.042
3	48 (10.0)	959 (13.3)	-3.3	0.74 [0.55–0.99]	0.042
4	48 (10.0)	962 (13.3)	-3.4	0.73 [0.55–0.98]	0.042
5	48 (10.0)	961 (13.3)	-3.3	0.74 [0.55–0.98]	0.042
6	48 (10.0)	959 (13.3)	-3.3	0.74 [0.55–0.99]	0.042
7	48 (10.0)	956 (13.3)	-3.3	0.74 [0.55–0.99]	0.042
8	48 (10.0)	959 (13.3)	-3.3	0.74 [0.55–0.99]	0.042
9	48 (10.0)	964 (13.4)	-3.4	0.73 [0.55–0.98]	0.042
10	48 (10.0)	956 (13.3)	-3.3	0.74 [0.55–0.99]	0.042

© 2021 Oskotsky T et al. *JAMA Network Open*.

eTable 8. Propensity Score (PS) Matching by Demographics, Encounter Type at the Time of the First Recorded COVID-19 Diagnosis, COVID-19 Comorbidities, and Prescription Indications for Other (Not Fluoxetine or Fluvoxamine) SSRI-Exposed Patients and Unexposed Control Patients

Iteration	Exposed to other SSRI, died, n (%), (n total = 2898)	Matched controls, died, n (%), (n total = 8694)	Difference in mortality rate (%)	RR [95% CI]	p-value (adjusted)
1	447 (15.4)	1479 (17.0)	-1.6	0.92 [0.84–1.00]	0.052
2	447 (15.4)	1489 (17.1)	-1.7	0.91 [0.83–0.99]	0.048
3	447 (15.4)	1482 (17.0)	-1.6	0.91 [0.84–1.00]	0.052
4	447 (15.4)	1501 (17.3)	-1.8	0.90 [0.83–0.99]	0.048
5	447 (15.4)	1496 (17.2)	-1.8	0.91 [0.83–0.99]	0.048
6	447 (15.4)	1491 (17.1)	-1.7	0.91 [0.83–0.99]	0.048
7	447 (15.4)	1496 (17.2)	-1.8	0.91 [0.83–0.99]	0.048
8	447 (15.4)	1474 (17.0)	-1.5	0.92 [0.84–1.00]	0.057
9	447 (15.4)	1494 (17.2)	-1.8	0.91 [0.83–0.99]	0.048
10	447 (15.4)	1493 (17.2)	-1.7	0.91 [0.83–0.99]	0.048

© 2021 Oskotsky T et al. *JAMA Network Open*.
